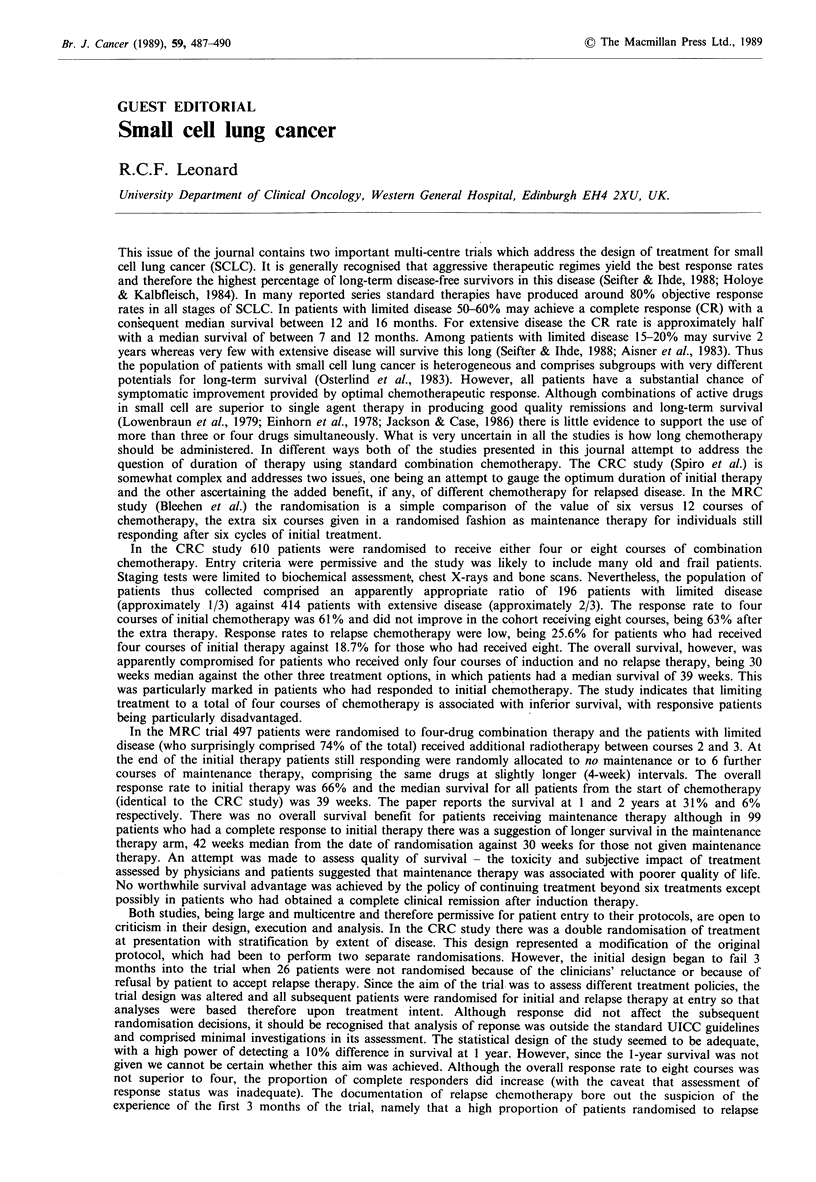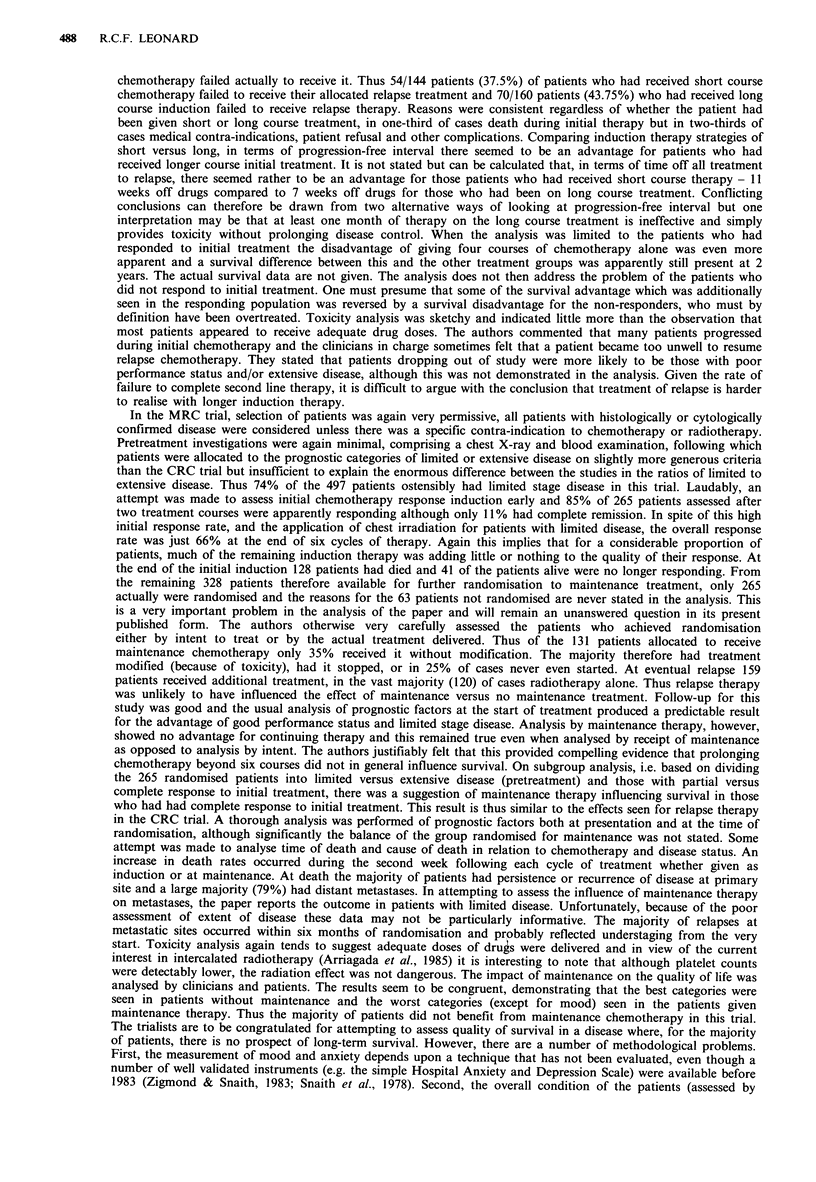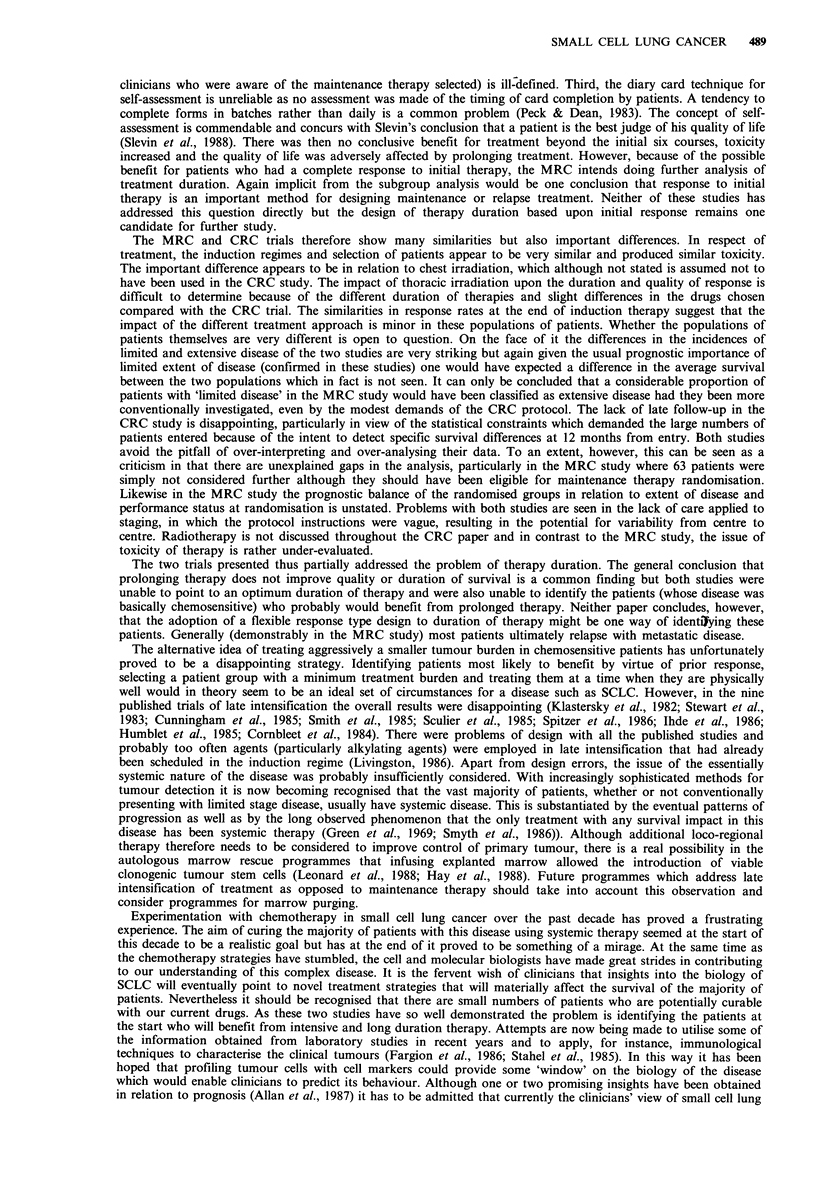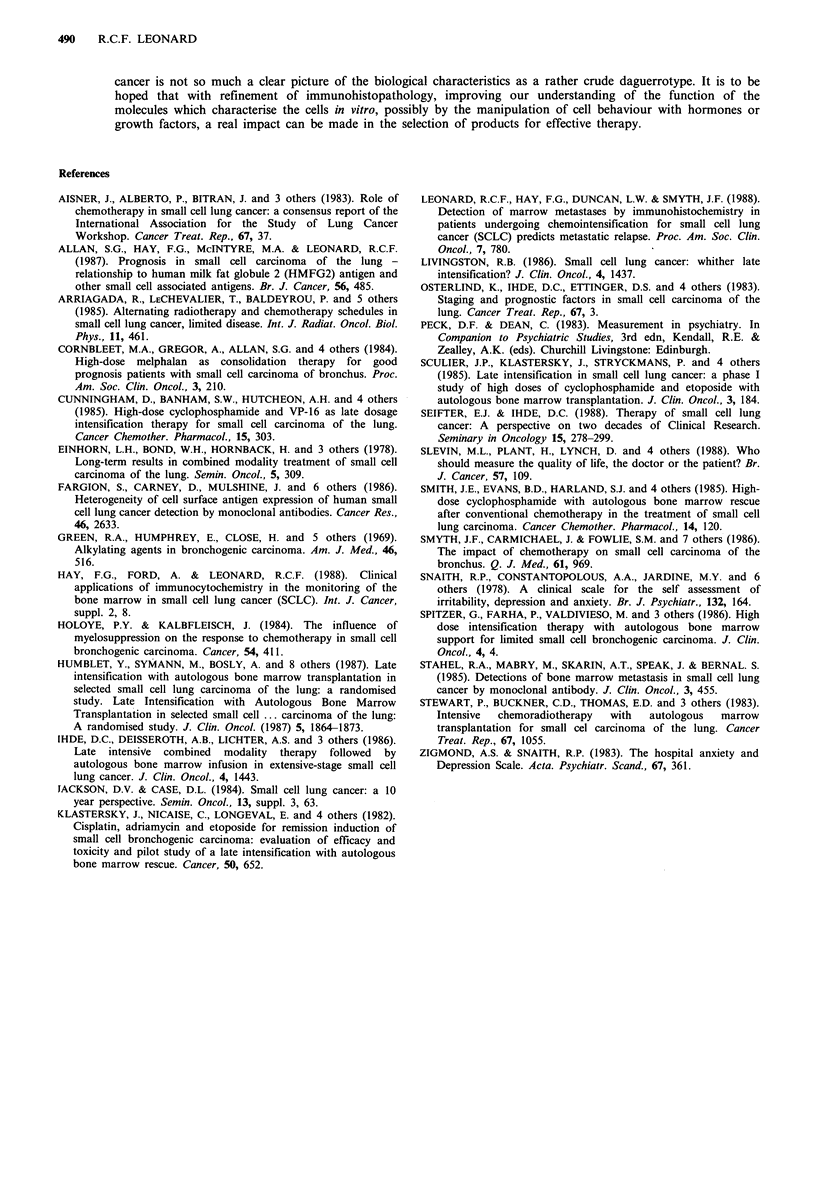# Small cell lung cancer.

**DOI:** 10.1038/bjc.1989.101

**Published:** 1989-04

**Authors:** R. C. Leonard

**Affiliations:** University Department of Clinical Oncology, Western General Hospital, Edinburgh, UK.


 © The Macmillan Press Ltd., 1989

GUEST EDITORIAL

### Small cell lung cancer

R.C.F. Leonard

*University Department of Clinical Oncology, Western General Hospital, Edinburgh EH4 2XU, UK.*

This issue of the journal contains two important multi-centre trials which address the design of treatment for small cell lung cancer (SCLC). It is generally recognised that aggressive therapeutic regimes yield the best response rates and therefore the highest percentage of long-term disease-free survivors in this disease (Seifter & Ihde, 1988; Holoye & Kalbfleisch, 1984). In many reported series standard therapies have produced around 80% objective response rates in all stages of SCLC. In patients with limited disease 50–60% may achieve a complete response (CR) with a consequent median survival between 12 and 16 months. For extensive disease the CR rate is approximately half with a median survival of between 7 and 12 months. Among patients with limited disease 15–20% may survive 2 years whereas very few with extensive disease will survive this long (Seifter & Ihde, 1988; Aisner *et al.*, 1983). Thus the population of patients with small cell lung cancer is heterogeneous and comprises subgroups with very different potentials for long-term survival (Osterlind *et al.*, 1983). However, all patients have a substantial chance of symptomatic improvement provided by optimal chemotherapeutic response. Although combinations of active drugs in small cell are superior to single agent therapy in producing good quality remissions and long-term survival (Lowenbraun *et al.*, 1979; Einhorn *et al.*, 1978; Jackson & Case, 1986) there is little evidence to support the use of more than three or four drugs simultaneously. What is very uncertain in all the studies is how long chemotherapy should be administered. In different ways both of the studies presented in this journal attempt to address the question of duration of therapy using standard combination chemotherapy. The CRC study (Spiro *et al.*) is somewhat complex and addresses two issues, one being an attempt to gauge the optimum duration of initial therapy and the other ascertaining the added benefit, if any, of different chemotherapy for relapsed disease. In the MRC study (Bleehen *et al.*) the randomisation is a simple comparison of the value of six versus 12 courses of chemotherapy, the extra six courses given in a randomised fashion as maintenance therapy for individuals still responding after six cycles of initial treatment.

In the CRC study 610 patients were randomised to receive either four or eight courses of combination chemotherapy. Entry criteria were permissive and the study was likely to include many old and frail patients. Staging tests were limited to biochemical assessment, chest X-rays and bone scans. Nevertheless, the population of patients thus collected comprised an apparently appropriate ratio of 196 patients with limited disease (approximately 1/3) against 414 patients with extensive disease (approximately 2/3). The response rate to four courses of initial chemotherapy was 61% and did not improve in the cohort receiving eight courses, being 63% after the extra therapy. Response rates to relapse chemotherapy were low, being 25.6% for patients who had received four courses of initial therapy against 18.7% for those who had received eight. The overall survival, however, was apparently compromised for patients who received only four courses of induction and no relapse therapy, being 30 weeks median against the other three treatment options, in which patients had a median survival of 39 weeks. This was particularly marked in patients who had responded to initial chemotherapy. The study indicates that limiting treatment to a total of four courses of chemotherapy is associated with inferior survival, with responsive patients being particularly disadvantaged.

In the MRC trial 497 patients were randomised to four-drug combination therapy and the patients with limited disease (who surprisingly comprised 74% of the total) received additional radiotherapy between courses 2 and 3. At the end of the initial therapy patients still responding were randomly allocated to *no* maintenance or to 6 further courses of maintenance therapy, comprising the same drugs at slightly longer (4-week) intervals. The overall response rate to initial therapy was 66% and the median survival for all patients from the start of chemotherapy (identical to the CRC study) was 39 weeks. The paper reports the survival at 1 and 2 years at 31% and 6% respectively. There was no overall survival benefit for patients receiving maintenance therapy although in 99 patients who had a complete response to initial therapy there was a suggestion of longer survival in the maintenance therapy arm, 42 weeks median from the date of randomisation against 30 weeks for those not given maintenance therapy. An attempt was made to assess quality of survival – the toxicity and subjective impact of treatment assessed by physicians and patients suggested that maintenance therapy was associated with poorer quality of life. No worthwhile survival advantage was achieved by the policy of continuing treatment beyond six treatments except possibly in patients who had obtained a complete clinical remission after induction therapy.

Both studies, being large and multicentre and therefore permissive for patient entry to their protocols, are open to criticism in their design, execution and analysis. In the CRC study there was a double randomisation of treatment at presentation with stratification by extent of disease. This design represented a modification of the original protocol, which had been to perform two separate randomisations. However, the initial design began to fail 3 months into the trial when 26 patients were not randomised because of the clinicians' reluctance or because of refusal by patient to accept relapse therapy. Since the aim of the trial was to assess different treatment policies, the trial design was altered and all subsequent patients were randomised for initial and relapse therapy at entry so that analyses were based therefore upon treatment intent. Although response did not affect the subsequent randomisation decisions, it should be recognised that analysis of reponse was outside the standard UICC guidelines and comprised minimal investigations in its assessment. The statistical design of the study seemed to be adequate, with a high power of detecting a 10% difference in survival at 1 year. However, since the 1-year survival was not given we cannot be certain whether this aim was achieved. Although the overall response rate to eight courses was not superior to four, the proportion of complete responders did increase (with the caveat that assessment of response status was inadequate). The documentation of relapse chemotherapy bore out the suspicion of the experience of the first 3 months of the trial, namely that a high proportion of patients randomised to relapse chemotherapy failed actually to receive it. Thus 54/144 patients (37.5%) of patients who had received short course chemotherapy failed to receive their allocated relapse treatment and 70/160 patients (43.75%) who had received long course induction failed to receive relapse therapy. Reasons were consistent regardless of whether the patient had been given short or long course treatment, in one-third of cases death during initial therapy but in two-thirds of cases medical contra-indications, patient refusal and other complications. Comparing induction therapy strategies of short versus long, in terms of progression-free interval there seemed to be an advantage for patients who had received longer course initial treatment. It is not stated but can be calculated that, in terms of time off all treatment to relapse, there seemed rather to be an advantage for those patients who had received short course therapy – 11 weeks off drugs compared to 7 weeks off drugs for those who had been on long course treatment. Conflicting conclusions can therefore be drawn from two alternative ways of looking at progression-free interval but one interpretation may be that at least one month of therapy on the long course treatment is ineffective and simply provides toxicity without prolonging disease control. When the analysis was limited to the patients who had responded to initial treatment the disadvantage of giving four courses of chemotherapy alone was even more apparent and a survival difference between this and the other treatment groups was apparently still present at 2 years. The actual survival data are not given. The analysis does not then address the problem of the patients who did not respond to initial treatment. One must presume that some of the survival advantage which was additionally seen in the responding population was reversed by a survival disadvantage for the non-responders, who must by definition have been overtreated. Toxicity analysis was sketchy and indicated little more than the observation that most patients appeared to receive adequate drug doses. The authors commented that many patients progressed during initial chemotherapy and the clinicians in charge sometimes felt that a patient became too unwell to resume relapse chemotherapy. They stated that patients dropping out of study were more likely to be those with poor performance status and/or extensive disease, although this was not demonstrated in the analysis. Given the rate of failure to complete second line therapy, it is difficult to argue with the conclusion that treatment of relapse is harder to realise with longer induction therapy.

In the MRC trial, selection of patients was again very permissive, all patients with histologically or cytologically confirmed disease were considered unless there was a specific contra-indication to chemotherapy or radiotherapy. Pretreatment investigations were again minimal, comprising a chest X-ray and blood examination, following which patients were allocated to the prognostic categories of limited or extensive disease on slightly more generous criteria than the CRC trial but insufficient to explain the enormous difference between the studies in the ratios of limited to extensive disease. Thus 74% of the 497 patients ostensibly had limited stage disease in this trial. Laudably, an attempt was made to assess initial chemotherapy response induction early and 85% of 265 patients assessed after two treatment courses were apparently responding although only 11% had complete remission. In spite of this high initial response rate, and the application of chest irradiation for patients with limited disease, the overall response rate was just 66% at the end of six cycles of therapy. Again this implies that for a considerable proportion of patients, much of the remaining induction therapy was adding little or nothing to the quality of their response. At the end of the initial induction 128 patients had died and 41 of the patients alive were no longer responding. From the remaining 328 patients therefore available for further randomisation to maintenance treatment, only 265 actually were randomised and the reasons for the 63 patients not randomised are never stated in the analysis. This is a very important problem in the analysis of the paper and will remain an unanswered question in its present published form. The authors otherwise very carefully assessed the patients who achieved randomisation either by intent to treat or by the actual treatment delivered. Thus of the 131 patients allocated to receive maintenance chemotherapy only 35% received it without modification. The majority therefore had treatment modified (because of toxicity), had it stopped, or in 25% of cases never even started. At eventual relapse 159 patients received additional treatment, in the vast majority (120) of cases radiotherapy alone. Thus relapse therapy was unlikely to have influenced the effect of maintenance versus no maintenance treatment. Follow-up for this study was good and the usual analysis of prognostic factors at the start of treatment produced a predictable result for the advantage of good performance status and limited stage disease. Analysis by maintenance therapy, however, showed no advantage for continuing therapy and this remained true even when analysed by receipt of maintenance as opposed to analysis by intent. The authors justifiably felt that this provided compelling evidence that prolonging chemotherapy beyond six courses did not in general influence survival. On subgroup analysis, i.e. based on dividing the 265 randomised patients into limited versus extensive disease (pretreatment) and those with partial versus complete response to initial treatment, there was a suggestion of maintenance therapy influencing survival in those who had had complete response to initial treatment. This result is thus similar to the effects seen for relapse therapy in the CRC trial. A thorough analysis was performed of prognostic factors both at presentation and at the time of randomisation, although significantly the balance of the group randomised for maintenance was not stated. Some attempt was made to analyse time of death and cause of death in relation to chemotherapy and disease status. An increase in death rates occurred during the second week following each cycle of treatment whether given as induction or at maintenance. At death the majority of patients had persistence or recurrence of disease at primary site and a large majority (79%) had distant metastases. In attempting to assess the influence of maintenance therapy on metastases, the paper reports the outcome in patients with limited disease. Unfortunately, because of the poor assessment of extent of disease these data may not be particularly informative. The majority of relapses at metastatic sites occurred within six months of randomisation and probably reflected understaging from the very start. Toxicity analysis again tends to suggest adequate doses of drugs were delivered and in view of the current interest in intercalated radiotherapy (Arriagada *et al.*, 1985) it is interesting to note that although platelet counts were detectably lower, the radiation effect was not dangerous. The impact of maintenance on the quality of life was analysed by clinicians and patients. The results seem to be congruent, demonstrating that the best categories were seen in patients without maintenance and the worst categories (except for mood) seen in the patients given maintenance therapy. Thus the majority of patients did not benefit from maintenance chemotherapy in this trial. The trialists are to be congratulated for attempting to assess quality of survival in a disease where, for the majority of patients, there is no prospect of long-term survival. However, there are a number of methodological problems. First, the measurement of mood and anxiety depends upon a technique that has not been evaluated, even though a number of well validated instruments (e.g. the simple Hospital Anxiety and Depression Scale) were available before 1983 (Zigmond & Snaith, 1983; Snaith *et al.*, 1978). Second, the overall condition of the patients (assessed by clinicians who were aware of the maintenance therapy selected) is ill-defined. Third, the diary card technique for self-assessment is unreliable as no assessment was made of the timing of card completion by patients. A tendency to complete forms in batches rather than daily is a common problem (Peck & Dean, 1983). The concept of self-assessment is commendable and concurs with Slevin's conclusion that a patient is the best judge of his quality of life (Slevin *et al.*, 1988). There was then no conclusive benefit for treatment beyond the initial six courses, toxicity increased and the quality of life was adversely affected by prolonging treatment. However, because of the possible benefit for patients who had a complete response to initial therapy, the MRC intends doing further analysis of treatment duration. Again implicit from the subgroup analysis would be one conclusion that response to initial therapy is an important method for designing maintenance or relapse treatment. Neither of these studies has addressed this question directly but the design of therapy duration based upon initial response remains one candidate for further study.

The MRC and CRC trials therefore show many similarities but also important differences. In respect of treatment, the induction regimes and selection of patients appear to be very similar and produced similar toxicity. The important difference appears to be in relation to chest irradiation, which although not stated is assumed not to have been used in the CRC study. The impact of thoracic irradiation upon the duration and quality of response is difficult to determine because of the different duration of therapies and slight differences in the drugs chosen compared with the CRC trial. The similarities in response rates at the end of induction therapy suggest that the impact of the different treatment approach is minor in these populations of patients. Whether the populations of patients themselves are very different is open to question. On the face of it the differences in the incidences of limited and extensive disease of the two studies are very striking but again given the usual prognostic importance of limited extent of disease (confirmed in these studies) one would have expected a difference in the average survival between the two populations which in fact is not seen. It can only be concluded that a considerable proportion of patients with 'limited disease' in the MRC study would have been classified as extensive disease had they been more conventionally investigated, even by the modest demands of the CRC protocol. The lack of late follow-up in the CRC study is disappointing, particularly in view of the statistical constraints which demanded the large numbers of patients entered because of the intent to detect specific survival differences at 12 months from entry. Both studies avoid the pitfall of over-interpreting and over-analysing their data. To an extent, however, this can be seen as a criticism in that there are unexplained gaps in the analysis, particularly in the MRC study where 63 patients were simply not considered further although they should have been eligible for maintenance therapy randomisation. Likewise in the MRC study the prognostic balance of the randomised groups in relation to extent of disease and performance status at randomisation is unstated. Problems with both studies are seen in the lack of care applied to staging, in which the protocol instructions were vague, resulting in the potential for variability from centre to centre. Radiotherapy is not discussed throughout the CRC paper and in contrast to the MRC study, the issue of toxicity of therapy is rather under-evaluated.

The two trials presented thus partially addressed the problem of therapy duration. The general conclusion that prolonging therapy does not improve quality or duration of survival is a common finding but both studies were unable to point to an optimum duration of therapy and were also unable to identify the patients (whose disease was basically chemosensitive) who probably would benefit from prolonged therapy. Neither paper concludes, however, that the adoption of a flexible response type design to duration of therapy might be one way of identifying these patients. Generally (demonstrably in the MRC study) most patients ultimately relapse with metastatic disease.

The alternative idea of treating aggressively a smaller tumour burden in chemosensitive patients has unfortunately proved to be a disappointing strategy. Identifying patients most likely to benefit by virtue of prior response, selecting a patient group with a minimum treatment burden and treating them at a time when they are physically well would in theory seem to be an ideal set of circumstances for a disease such as SCLC. However, in the nine published trials of late intensification the overall results were disappointing (Klastersky *et al.*, 1982; Stewart *et al.*, 1983; Cunningham *et al.*, 1985; Smith *et al.*, 1985; Sculier *et al.*, 1985; Spitzer *et al.*, 1986; Ihde *et al.*, 1986; Humblet *et al.*, 1985; Cornbleet *et al.*, 1984). There were problems of design with all the published studies and probably too often agents (particularly alkylating agents) were employed in late intensification that had already been scheduled in the induction regime (Livingston, 1986). Apart from design errors, the issue of the essentially systemic nature of the disease was probably insufficiently considered. With increasingly sophisticated methods for tumour detection it is now becoming recognised that the vast majority of patients, whether or not conventionally presenting with limited stage disease, usually have systemic disease. This is substantiated by the eventual patterns of progression as well as by the long observed phenomenon that the only treatment with any survival impact in this disease has been systemic therapy (Green *et al.*, 1969; Smyth *et al.*, 1986)). Although additional loco-regional therapy therefore needs to be considered to improve control of primary tumour, there is a real possibility in the autologous marrow rescue programmes that infusing explanted marrow allowed the introduction of viable clonogenic tumour stem cells (Leonard *et al.*, 1988; Hay *et al.*, 1988). Future programmes which address late intensification of treatment as opposed to maintenance therapy should take into account this observation and consider programmes for marrow purging.

Experimentation with chemotherapy in small cell lung cancer over the past decade has proved a frustrating experience. The aim of curing the majority of patients with this disease using systemic therapy seemed at the start of this decade to be a realistic goal but has at the end of it proved to be something of a mirage. At the same time as the chemotherapy strategies have stumbled, the cell and molecular biologists have made great strides in contributing to our understanding of this complex disease. It is the fervent wish of clinicians that insights into the biology of SCLC will eventually point to novel treatment strategies that will materially affect the survival of the majority of patients. Nevertheless it should be recognised that there are small numbers of patients who are potentially curable with our current drugs. As these two studies have so well demonstrated the problem is identifying the patients at the start who will benefit from intensive and long duration therapy. Attempts are now being made to utilise some of the information obtained from laboratory studies in recent years and to apply, for instance, immunological techniques to characterise the clinical tumours (Fargion *et al.*, 1986; Stahel *et al.*, 1985). In this way it has been hoped that profiling tumour cells with cell markers could provide some 'window' on the biology of the disease which would enable clinicians to predict its behaviour. Although one or two promising insights have been obtained in relation to prognosis (Allan *et al.*, 1987) it has to be admitted that currently the clinicians' view of small cell lung cancer is not so much a clear picture of the biological characteristics as a rather crude daguerrotype. It is to be hoped that with refinement of immunohistopathology, improving our understanding of the function of the molecules which characterise the cells *in vitro*, possibly by the manipulation of cell behaviour with hormones or growth factors, a real impact can be made in the selection of products for effective therapy.

### References

AISNER, J., ALBERTO, P., BITRAN, J. and 3 others (1983). Role of chemotherapy in small cell lung cancer: a consensus report of the International Association for the Study of Lung Cancer Workshop. *Cancer Treat. Rep.*, **67**, 37.

ALLAN, S.G., HAY, F.G., McINTYRE, M.A. & LEONARD, R.C.F. (1987). Prognosis in small cell carcinoma of the lung – relationship to human milk fat globule 2 (HMFG2) antigen and other small cell associated antigens. *Br. J. Cancer*, **56**, 485.

ARRIAGADA, R., LECHEVALIER, T., BALDEYROU, P. and 5 others (1985). Alternating radiotherapy and chemotherapy schedules in small cell lung cancer, limited disease. *Int. J. Radiat. Oncol. Biol. Phys.*, **11**, 461.

CORNBLEET, M.A., GREGOR, A., ALLAN, S.G. and 4 others (1984). High-dose melphalan as consolidation therapy for good prognosis patients with small cell carcinoma of bronchus. *Proc. Am. Soc. Clin. Oncol.*, **3**, 210.

CUNNINGHAM, D., BANHAM, S.W., HUTCHEON, A.H. and 4 others (1985). High-dose cyclophosphamide and VP-16 as late dosage intensification therapy for small cell carcinoma of the lung. *Cancer Chemother. Pharmacol.*, **15**, 303.

EINHORN, L.H., BOND, W.H., HORNBACK, H. and 3 others (1978). Long-term results in combined modality treatment of small cell carcinoma of the lung. *Semin. Oncol.*, **5**, 309.

FARGION, S., CARNEY, D., MULSHINE, J. and 6 others (1986). Heterogeneity of cell surface antigen expression of human small cell lung cancer detection by monoclonal antibodies. *Cancer Res.*, **46**, 2633.

GREEN, R.A., HUMPHREY, E., CLOSE, H. and 5 others (1969). Alkylating agents in bronchogenic carcinoma. *Am. J. Med.*, **46**, 516.

HAY, F.G., FORD, A. & LEONARD, R.C.F. (1988). Clinical applications of immunocytochemistry in the monitoring of the bone marrow in small cell lung cancer (SCLC). *Int. J. Cancer*, suppl. 2, 8.

HOLOYE, P.Y. & KALBFLEISCH, J. (1984). The influence of myelosuppression on the response to chemotherapy in small cell bronchogenic carcinoma. *Cancer*, **54**, 411.

HUMBLET, Y., SYMANN, M., BOSLY, A. and 8 others (1987). Late intensification with autologous bone marrow transplantation in selected small cell lung carcinoma of the lung: a randomised study. Late Intensification with Autologous Bone Marrow Transplantation in selected small cell … carcinoma of the lung: A randomised study. *J. Clin. Oncol.* (1987) **5**, 1864–1873.

IHDE, D.C., DEISSEROTH, A.B., LICHTER, A.S. and 3 others (1986). Late intensive combined modality therapy followed by autologous bone marrow infusion in extensive-stage small cell lung cancer. *J. Clin. Oncol.*, **4**, 1443.

JACKSON, D.V. & CASE, D.L. (1984). Small cell lung cancer: a 10 year perspective. *Semin. Oncol.*, **13**, suppl. 3, 63.

KLASTERSKY, J., NICAISE, C., LONGEVAL, E. and 4 others (1982). Cisplatin, adriamycin and etoposide for remission induction of small cell bronchogenic carcinoma: evaluation of efficacy and toxicity and pilot study of a late intensification with autologous bone marrow rescue. *Cancer*, **50**, 652.

LEONARD, R.C.F., HAY, F.G., DUNCAN, L.W. & SMYTH, J.F. (1988). Detection of marrow metastases by immunohistochemistry in patients undergoing chemointensification for small cell lung cancer (SCLC) predicts metastatic relapse. *Proc. Am. Soc. Clin. Oncol.*, **7**, 780.

LIVINGSTON, R.B. (1986). Small cell lung cancer: whither late intensification? *J. Clin. Oncol.*, **4**, 1437.

OSTERLIND, K., IHDE, D.C., ETTINGER, D.S. and 4 others (1983). Staging and prognostic factors in small cell carcinoma of the lung. *Cancer Treat. Rep.*, **67**, 3.

PECK, D.F. & DEAN, C. (1983). Measurement in psychiatry. In *Companion to Psychiatric Studies*, 3rd edn, Kendall, R.E. & Zealley, A.K. (eds). Churchill Livingstone: Edinburgh.

SCULIER, J.P., KLASTERSKY, J., STRYCKMANS, P. and 4 others (1985). Late intensification in small cell lung cancer: a phase I study of high doses of cyclophosphamide and etoposide with autologous bone marrow transplantation. *J. Clin. Oncol.*, **3**, 184.

SEIFTER, E.J. & IHDE, D.C. (1988). Therapy of small cell lung cancer: A perspective on two decades of Clinical Research. *Seminary in Oncology* **15**, 278–299.

SLEVIN, M.L., PLANT, H., LYNCH, D. and 4 others (1988). Who should measure the quality of life, the doctor or the patient? *Br. J. Cancer*, **57**, 109.

SMITH, J.E., EVANS, B.D., HARLAND, S.J. and 4 others (1985). High-dose cyclophosphamide with autologous bone marrow rescue after conventional chemotherapy in the treatment of small cell lung carcinoma. *Cancer Chemother. Pharmacol.*, **14**, 120.

SMYTH, J.F., CARMICHAEL, J. & FOWLIE, S.M. and 7 others (1986). The impact of chemotherapy on small cell carcinoma of the bronchus. *Q. J. Med.*, **61**, 969.

SNAITH, R.P., CONSTANTOPOLOUS, A.A., JARDINE, M.Y. and 6 others (1978). A clinical scale for the self assessment of irritability, depression and anxiety. *Br. J. Psychiatr.*, **132**, 164.

SPITZER, G., FARHA, P., VALDIVIESO, M. and 3 others (1986). High dose intensification therapy with autologous bone marrow support for limited small cell bronchogenic carcinoma. *J. Clin. Oncol.*, **4**, 4.

STAHEL, R.A., MABRY, M., SKARIN, A.T., SPEAK, J. & BERNAL. S. (1985). Detections of bone marrow metastasis in small cell lung cancer by monoclonal antibody. *J. Clin. Oncol.*, **3**, 455.

STEWART, P., BUCKNER, C.D., THOMAS, E.D. and 3 others (1983). Intensive chemoradiotherapy with autologous marrow transplantation for small cel carcinoma of the lung. *Cancer Treat. Rep.*, **67**, 1055.

ZIGMOND, A.S. & SNAITH, R.P. (1983). The hospital anxiety and Depression Scale. *Acta. Psychiatr. Scand.*, **67**, 361.